# A transcriptional regulatory network of *Rsv3*-mediated extreme resistance against *Soybean mosaic virus*

**DOI:** 10.1371/journal.pone.0231658

**Published:** 2020-04-21

**Authors:** Lindsay C. DeMers, Neelam R. Redekar, Aardra Kachroo, Sue A. Tolin, Song Li, M. A. Saghai Maroof

**Affiliations:** 1 School of Plant and Environmental Sciences, Virginia Tech, Blacksburg, Virginia, United States of America; 2 Department of Plant Pathology, University of Kentucky, Lexington, Virginia, United States of America; Texas A&M University System, UNITED STATES

## Abstract

Resistance genes are an effective means for disease control in plants. They predominantly function by inducing a hypersensitive reaction, which results in localized cell death restricting pathogen spread. Some resistance genes elicit an atypical response, termed extreme resistance, where resistance is not associated with a hypersensitive reaction and its standard defense responses. Unlike hypersensitive reaction, the molecular regulatory mechanism(s) underlying extreme resistance is largely unexplored. One of the few known, naturally occurring, instances of extreme resistance is resistance derived from the soybean *Rsv3* gene, which confers resistance against the most virulent *Soybean mosaic virus* strains. To discern the regulatory mechanism underlying *Rsv3*-mediated extreme resistance, we generated a gene regulatory network using transcriptomic data from time course comparisons of *Soybean mosaic virus*-G7-inoculated resistant (L29, *Rsv3*-genotype) and susceptible (Williams82, *rsv3*-genotype) soybean cultivars. Our results show *Rsv3* begins mounting a defense by 6 hpi via a complex phytohormone network, where abscisic acid, cytokinin, jasmonic acid, and salicylic acid pathways are suppressed. We identified putative regulatory interactions between transcription factors and genes in phytohormone regulatory pathways, which is consistent with the demonstrated involvement of these pathways in *Rsv3*-mediated resistance. One such transcription factor identified as a putative transcriptional regulator was MYC2 encoded by Glyma.07G051500. Known as a master regulator of abscisic acid and jasmonic acid signaling, MYC2 specifically recognizes the G-box motif (“CACGTG”), which was significantly enriched in our data among differentially expressed genes implicated in abscisic acid- and jasmonic acid-related activities. This suggests an important role for Glyma.07G051500 in abscisic acid- and jasmonic acid-derived defense signaling in *Rsv3*. Resultantly, the findings from our network offer insights into genes and biological pathways underlying the molecular defense mechanism of *Rsv3*-mediated extreme resistance against *Soybean mosaic virus*. The computational pipeline used to reconstruct the gene regulatory network in this study is freely available at https://github.com/LiLabAtVT/rsv3-network.

## Introduction

Soybean is a crop of global importance, and the *Soybean mosaic virus* (SMV)-soybean pathosystem provides an opportunity to study the extreme resistance (ER) response, a type of resistance unique from the typical hypersensitive reaction (HR) response in that it is triggered earlier and cell death is not observed [1]. SMV, a single-stranded RNA virus of the genus *Potyvirus*, considerably reduces seed quality and yield in soybean-growing regions throughout the world. Several SMV isolates recovered from germplasm imported into the United States were classified into seven strain groups, G1 to G7, based on reactions in a set of various soybean genotypes [2]. The most successful management strategies have been the utilization of virus-free seeds and resistant cultivars carrying resistance (*R*) genes. Four dominant *R* genes have been identified—*Rsv1*, *Rsv3*, *Rsv4*, and *Rsv5* [3–8]. *Rsv1* and *Rsv3* confer ER against SMV strains G1 to G4 and G5 to G7, respectively [5, 9, 10]. Among these strains, G5 to G7 represent the most virulent SMV strains, making *Rsv3* a particularly interesting gene for functional study. The *Rsv3* locus has been mapped, and the gene responsible for conditioning *Rsv3*-mediated resistance (Glyma.14g204700; Glyma.Wm82.a2.v1 gene model) has been identified [11–13]. Comparative sequence analysis has revealed that Glyma.14g204700 is highly polymorphic in the LRR domain of soybean lines carrying *Rsv3*. This suggests *Rsv3*-mediated resistance is initiated by the LRR domain’s recognition of an effector, the SMV cylindrical inclusion protein (CI) [12, 14]. However, the events directly following recognition remain undefined. It is hypothesized in [15] that the abscisic acid (ABA) signaling pathway is triggered during later stages of the *Rsv3*-mediated ER response. The consequent high ABA levels induce expression of a family of type 2C protein phosphatases, resulting in callose deposition, which impedes viral cell-to-cell movement [15]. Nonetheless, a large gap remains in our understanding of the *Rsv3*-mediated ER response, as the initial molecular events occurring prior to activation of the ABA signaling pathway are still unknown.

One approach to discerning the underlying mechanisms controlling a biological process, such as in *Rsv3*-mediated resistance, is reconstructing and modeling its molecular network. These networks examine complex interactions between genes, proteins, and metabolites. At the gene level, expression is predominantly governed by transcription factors (TFs), which bind to DNA sequence motifs in the regulatory region of their target genes. Improved understanding of gene expression regulation can have considerable scientific impact as many of the biological control mechanisms responsible for certain traits are associated with mutations in regulatory regions or dysfunctional transcriptional regulators [16]. For example, modern-day crops such as maize, rice, and wheat were heavily shaped by alterations in transcriptional regulation [17]; accordingly, elucidation of transcriptional regulation can aid significantly in research. An approach to accomplish this is the utilization of gene regulatory networks (GRNs), the study of which has led to the discovery of important genes and regulatory mechanisms underlying specific processes in *Escherichia coli*, *Saccharomyces cerevisiae*, and *Arabidopsis thaliana* [18–23]. GRNs describe the intricate web of TFs that bind regulatory regions of target genes in order to influence their spatial and temporal expression [24]. Using computational network inference methods, the structure of the gene regulatory interactions that makeup GRNs can be reverse-engineered. That is, causal relationships can be inferred between genes (such as those encoding TFs) directly controlling the expression of other genes [25, 26]. By taking advantage of advancements in high-throughput sequencing technology, GRNs can be reconstructed utilizing genome-wide expression data, such as from RNA sequencing (RNA-seq) [27]. RNA-seq analyses can identify thousands of genes with altered expression in response to virus inoculation and provide more molecular targets to study. Network inference methods can then be applied to the expression data to uncover key genes and regulatory relationships [16]. Thus, the significance of modeling transcriptional regulation is that it provides a means for discerning gene function and important regulators in molecular pathways, such as those involved in mediating the *Rsv3*-mediated ER response.

This study aims to elucidate the key regulatory components involved in the *Rsv3* defense mechanism by constructing a GRN. To do this, we performed a comparative transcriptomic time course analysis of SMV-G7-inoculated cultivars “L29” (*Rsv3*-genotype) and “Williams82” (*rsv3*-genotype) during the early hours post-inoculation. We found differentially expressed genes (DEGs) between L29 and Williams82 at each time point, and among these were several genes belonging to TF families associated with defense. We carried out GRN inference analyses on DEGs utilizing the computational pipeline we developed previously [28]. This pipeline makes use of the well-received module networks method in which GRNs are inferred between TFs and gene co-expression modules. Network inference was performed with unique unsupervised learning algorithms: ARACNE (Algorithm for the Reconstruction of Accurate Cellular Networks), context likelihood of relatedness (CLR), least angle regression (LARS), partial correlation, and Random Forest [29–33]. These algorithms represent the top performing inference methods according to the DREAM5 benchmark challenge [34]. Several of the predicted interactions were validated using published interactions in the model plant species, *A*. *thaliana*, and by motif sequence analysis [35–37].

## Materials and methods

### *Soybean mosaic virus* inoculations, leaf sampling, and RNA extraction

For this study, we used SMV strain G7 (SMV-G7) inoculum originating from [2]. The inoculum was stored in the form of desiccated infected leaves for long-term storage at 5°C or frozen at -80°C. Response of differential cultivars for “trueness to type” was tested periodically as inoculum were activated from storage. In this study, the SMV-G7 strain was maintained on greenhouse-grown soybean cultivar “York” (*rsv3*-genotype “susceptible”) prior to the experiment. The SMV-G7 inoculum was prepared from symptomatic trifoliolate leaves of York by crushing in a mortar and pestle with 0.01M sodium phosphate buffer–pH 7.0 (1:10 w/v). The inoculation experiment was performed in greenhouse in the spring of 2014, where temperature, humidity, and light conditions were not artificially controlled. Inoculations were performed by lightly dusting 600-mesh carborundum powder over unifoliolate leaves, and the virus inoculum (see above) was gently rubbed using a pestle onto the two unifoliolate leaves of each plant and followed by a gentle rinsing with tap water. The inoculated unifoliolate leaves were collected at 0, 2, 4, 6, and 8 hours post inoculation (hpi) in biological triplicate, rinsed with DI water, frozen immediately by immersing in liquid nitrogen, and stored at -80°C until RNA extraction. For each time point, a single biological replicate sample was comprised of six unifoliolate leaves total (= 2 unifoliolate leaves per plant x 3 individual plants within a pot). Thus 15 plants (= 3 plants per time point x 5 time points) were sampled from both cultivars. Total RNA (RIN >7.0) was extracted from frozen samples using RNeasy Plant Mini Kit (QIAGEN, Hilden, Germany) with on-column DNase digestion (QIAGEN, Hilden, Germany). A total of 20 mRNA libraries (= 2 cultivars x 5 time points x 2 biological replicates) was prepared from duplicate RNA samples of each virus-inoculated cultivar at each time point and sequenced as 150 PE with Illumina HiSeq4000 (Illumina, San Diego, CA) at Novogene, Sacramento, CA.

### Sequence data processing and differential gene expression

Raw reads were filtered using Trimmomatic (version 0.30) to remove adapter sequences (ILLUMINACLIP:<IlluminaAdapters.fa>:2:30:10), trim low quality bases (<Q30, LEADING:30 TRAILING:30), and remove those reads trimmed to less than 50 base pairs (MINLEN:50) [38]. Reads were mapped to the “Williams82” soybean reference genome (Wm82.a2.v1, downloaded from Phytozome) using STAR (version 2.5.3a) with a maximum intron length of 15000 (--alignIntronMax) [39, 40]. The number of reads mapped to each gene was quantified using featureCounts (version 1.5.3) using paired end parameters “-B” and “-p” with features defined as “exons” (-t) being grouped by “gene_id” (-g) [41]. Differential expression analysis was performed with DESeq2 (version 1.22.2) in R (version 3.5.1) with those genes having less than one count being removed [42]. Reference levels were set as the susceptible Williams82 line and 0 hpi, and the DESeq() function “test” parameter was set to “LRT”. The resulting output was used to make comparisons between L29 and Williams82 to identify DEGs at each time point by employing the results() function with the “test” parameter set as “Wald”. DEGs were defined as those with a false discovery rate (FDR) adjusted p-value < 0.05, log_2_ fold change >|1.0|, and base mean >10. DEGs and their log_2_ fold changes can be found in S1 Table. The RNA-seq data from this study are available at the NCBI Gene Expression Omnibus (GEO) repository under accession number GSE137263.

### Inference of gene regulatory networks

#### Expression clustering and gene function annotation

Gene expression levels for all genes were normalized by variance-stabilizing transformation in DESeq2 and averaged across replicates [42]. Clustering analysis was carried out on DEGs using Gaussian-finite mixture modeling with the R package, mclust (version 5.4.2) using default parameters [43]. The optimal clustering model was determined using Bayesian Information Criteria (BIC) and integrated complete-data likelihood (ICL) criterion [44, 45]. The top performing model identified five gene clusters. Gene ontology (GO) enrichment analysis was performed on each gene cluster using soybean GO annotations from [46]. Significantly enriched GO categories were selected using Fisher’s exact test with FDR <0.05 (S2 Table) Significantly enriched gene families were also analyzed using GenFam online tool, and the results with FDR <0.05 are included (S2 Table) [47]. DEGs encoding TFs were identified using TF annotations downloaded from PlantTFDB [48].

#### Network inference methods

Network inference was carried out following the pipeline we developed previously using machine learning methods [28]. Gaussian-finite mixture modeling was used to cluster DEGs, with the best model finding five clusters (gene modules). We identified 131 differentially expressed TFs, which were set as putative regulators of the five modules. The mean expression profile for each module was computed and then constructed into an expression matrix of these values and the expression levels of the 131 TFs. Putative regulatory interactions between each TF and gene module were inferred from the expression matrix by implementing five unique network inference algorithms: ARACNE, CLR, LARS, partial correlation, and Random Forest [29–33]. ARACNE and CLR inference methods were implemented with the R package minet (version 3.40.0) with the “estimator” parameter set as “spearman” and the “eps” parameter set as 0.1 for ARACNE and for CLR the “estimator” set as “pearson” [30, 31, 49]. The LARS inference method was implemented with the R package tigress (version 0.1.0) with “nstepsLARS” set at 4 [33]. The partial correlation inference method was implemented with the R package GeneNet (1.2.13) using the “dynamic” shrinkage method [29, 50]. Lastly, the Random Forest inference method was implemented with the R package GENIE3 (version 1.4.3) with all default parameters [32]. Because community-based approaches make for a more robust inference of GRNs, multiple inference methods, based on a diverse set of algorithms, were applied to predict interactions. These methods were among the top performing in the DREAM5 challenge [34].

#### Validation of inferred network interactions

We used two approaches to validate the discovered putative regulatory interactions predicted by the inference methods. The first approach entailed the identification of homologous regulatory interactions in *A*. *thaliana* using a comprehensive set of published *A*. *thaliana* interactions observed with DNA affinity purification sequencing (DAP-seq) [35]. This DAP-seq dataset is composed of 2.8 million interactions between 387 TFs and 32,605 genes. For comparison of our predicted regulatory network with the *A*. *thaliana* DAP-seq data, we first expanded the TF-module interactions to TF-gene interactions. That is, each TF was set as a putative regulator of all the genes in the modules it was predicted to regulate. Homologous *A*. *thaliana* interactions for the TF-gene interactions were generated by using BLAST to identify *A*. *thaliana* homologous genes with soybean gene coding sequences. The best one-to-one BLAST hits were selected, using an E-value of 1e-5 for cut off. The resulting homologous *A*. *thaliana* interactions were then compared to the DAP-seq dataset and matching interactions identified.

For the second method of network validation, we performed motif sequence analysis using Meme suite (version 5.0.4), which provides a set of tools for motif discovery, enrichment, scanning, and comparison [36]. With this approach, we identified putative TF binding sites in promoter regions (defined as the 1000 bps flanking a gene’s 5’ end) of the DEGs in each module. These binding sites (motifs) were identified using the motif discovery tool, MEME [37]. The TomTom tool was then used to compare the discovered motif sequences to 872 *A*. *thaliana* motifs found with DAP-seq and to identify TFs that may bind to those discovered sequences [35, 51].

## Results and discussion

In this study, we analyzed the transcriptional regulation of the *R* gene *Rsv3*, which confers ER against the most virulent SMV strains. This was accomplished by implementing machine learning inference algorithms on a GRN constructed from time course RNA-seq data from leaves of SMV-G7 inoculated resistant and susceptible soybean cultivars, L29 and Williams82, respectively. Our results suggest that an intricate regulatory network is in place modulating the *Rsv3*-mediated resistance response upon SMV-G7 inoculation.

### Fate of SMV-induced susceptibility or resistance in soybean is determined between 4 to 8 hours post-inoculation

**Fig 1:**
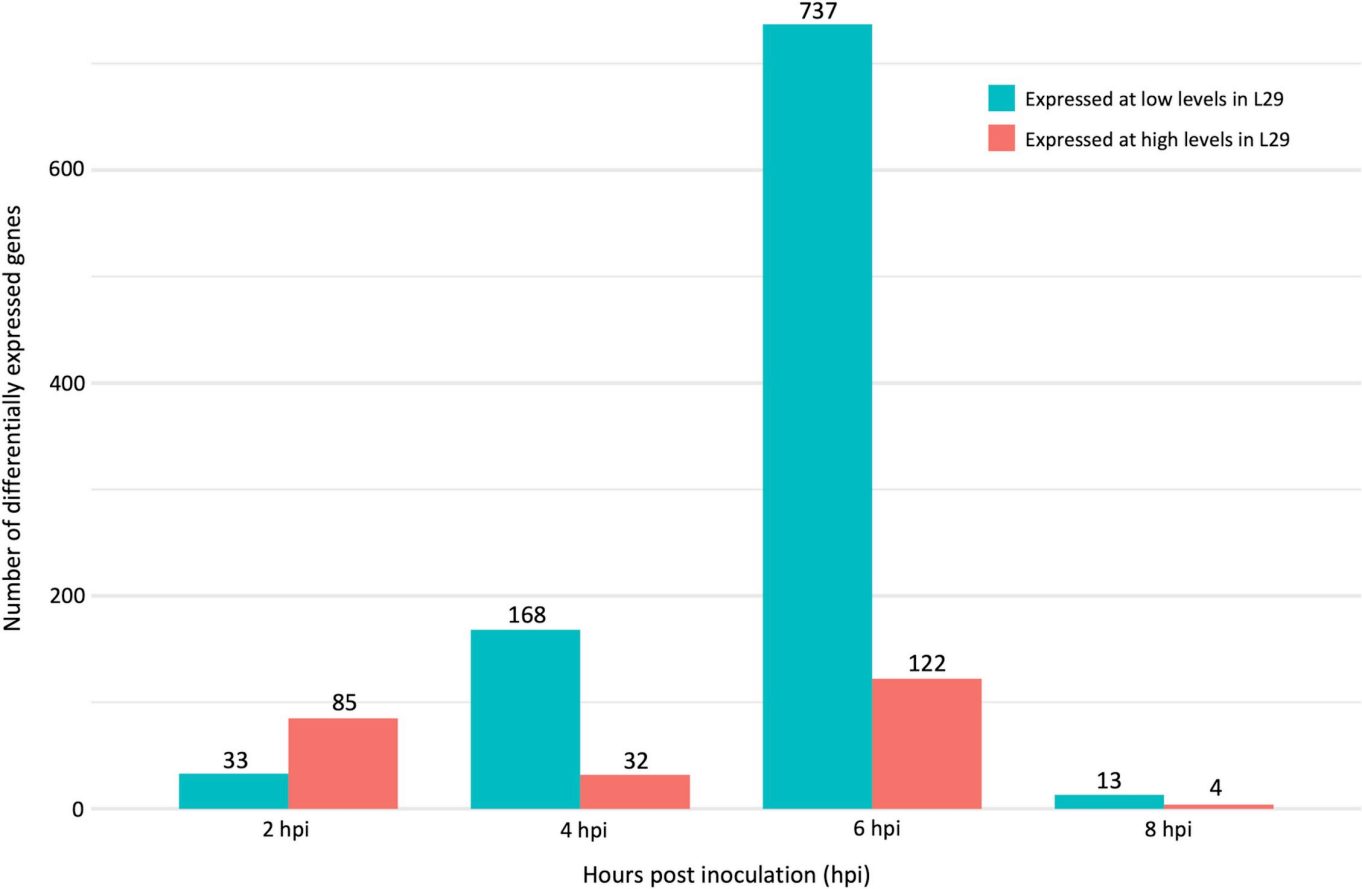
Number of differentially expressed genes between soybean cultivars L29 and Williams82 at 2, 4, 6, and 8 hours post inoculation with *Soybean mosaic virus* strain G7. DEGs were defined as those with FDR adjusted p-value < 0.05, log_2_ fold change >|1.0|, and base mean >10. High expression or low expression in L29 means the expression of DEG was either higher or lower in L29 as compared to Williams82, respectively. A total of 1128 DEGs were identified between L29 and Williams82 at 2, 4, 6 and 8 hpi. DEGs at 0 hpi were minimal and excluded, being considered effects of differences in genetic backgrounds of the two cultivars and not infection responses.

To better understand the regulatory mechanism underlying *Rsv3*-mediated ER, we compared transcriptomic profiles of SMV-G7 inoculated leaves from L29 and Williams82 cultivars at 0, 2, 4, 6 and 8 hpi. Overall, 1128 genes were differentially expressed between two cultivars, at one or more time points between 2 and 8 hpi (S1 Table); DEGs identified at 0 hpi were excluded, as they were considered effects from differences in genetic backgrounds between the two cultivars. Distribution of the 1128 DEGs found between 2 and 8 hpi is shown in Fig 1. The majority of transcriptomic changes occurred between 4 and 8 hpi, suggesting that the large shifts in transcriptional activity during this time frame may be critical to whether a susceptible or defense response is induced. There was a striking increase in the number of DEGs at 6 hpi (859 DEGs), accounting for more than 75% of the total number of DEGs. This was followed by a dramatic drop at 8 hpi to merely 17 DEGs. This likely implies the presence of a tightly defined regulatory system that elicits the *Rsv3*-mediated ER response, suggesting the *Rsv3* pathway is induced very early during the infection process and that a susceptible or resistant response to SMV may be determined by 6 hpi.

At 6 hpi, GO enrichment analyses revealed that the 122 DEGs highly expressed in L29 were involved in cytokinin metabolism and signaling. Also highly expressed was a unique subfamily of MYB-related TFs, the RADIALIS-LIKE SANT/MYBs (RSMs). Up-regulation of six differentially expressed members of this family, specifically at 6 hpi, suggests tight temporal regulation of RSM TFs, which could be important to a process essential in ER-mediated defense. Little is known about the RSM subfamily, but one study showed involvement of RSM1 in auxin signaling [52]. No other TF family was exclusively highly expressed or had multiple members up-regulated at this time. Interestingly, more than 85% of the DEGs in this time period (4–8 hpi) were expressed at lower levels in L29 as compared to Williams82. At 6 hpi, most of the down-regulated genes were those responsive to water deprivation, light absence, sucrose starvation, genes encoding stress-related proteins, such as multiple glutathione S-transferases, heat shock and LEA (late embryogenesis abundant) chaperones, and proteins related to oxidative stress and signaling, such as transporters, serine/threonine kinases, and receptor kinases. Additionally, a number of genes in the ABA signaling and the salicylic acid (SA) pathways were down-regulated in L29 as well. This finding is unique in that the activation of the SA pathway and exogenous application of SA are both widely recognized as enhancing resistance to viruses [53]. Nevertheless, a few exceptions to this phenomenon have been observed; in inoculated and systemically infected leaves of soybean, SA treatment had no effect on *Bean pod mottle virus* (BPMV) accumulation, and in susceptible pea cultivars, activation of the SA pathway resulted in an increase of *Clover yellow vein virus* virulence [54, 55]. Nonetheless, it remains unclear how SA, in some cases, enhances virulence [53], suggesting that suppression of the SA pathway may be a facet of *Rsv3’*s mechanism for diverting SMV-G7 infection.

### Biological processes associated with *Rsv3*-mediated resistance in soybean show differential hormone responses

**Fig 2:**
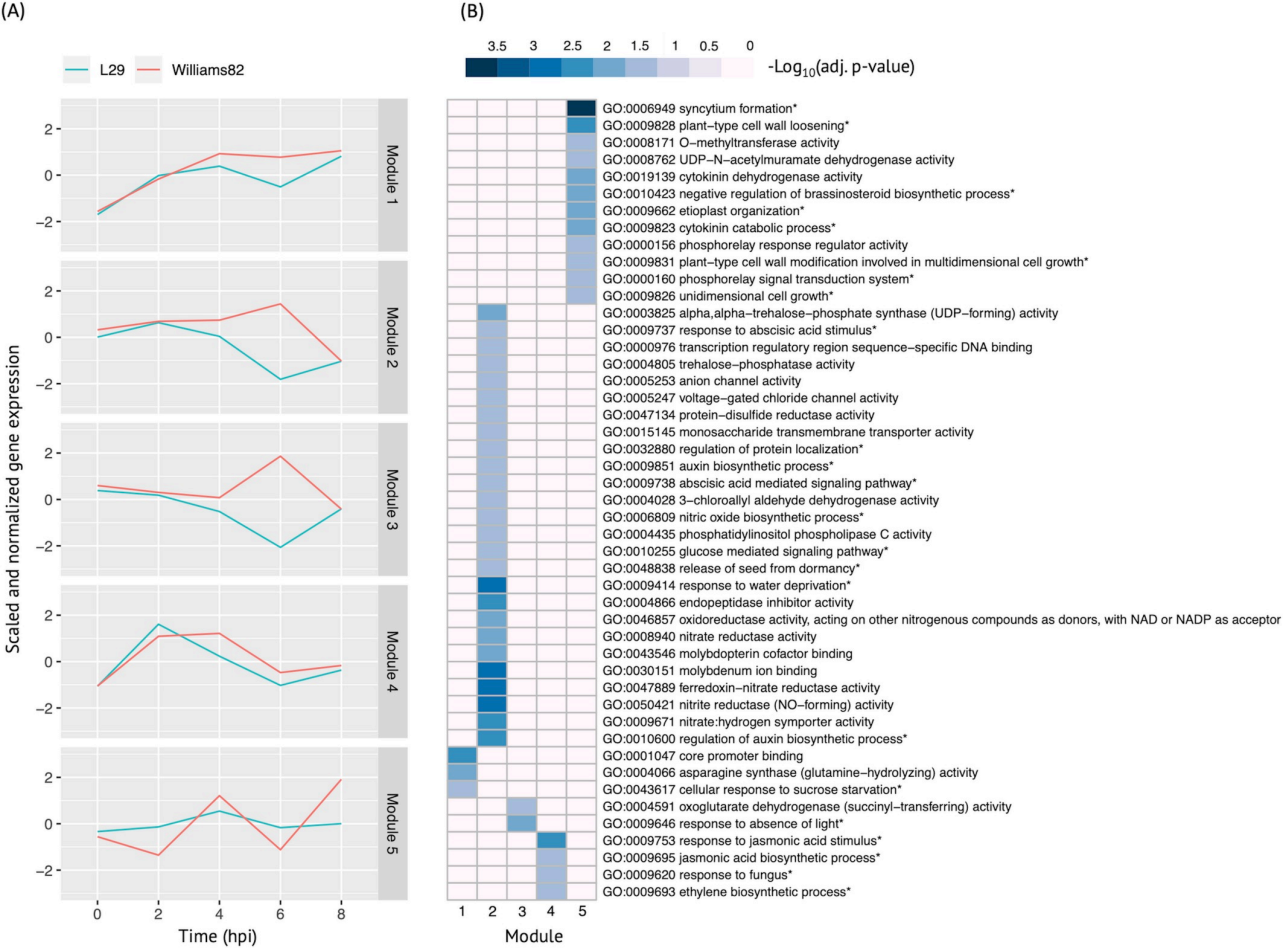
Co-expression gene modules and their biological functions. A module is defined as a group of genes sharing similar expression profiles over time and likely involved in the same biological processes. The expression profile for these modules was determined by averaging the expression levels of DEGs within each module. (A) Mean module expression profiles of L29 and Williams82 over time. Normalized expressions of DEGs were used for clustering with Gaussian-finite mixture modeling. (B) Heatmap of GO functional enrichment analyses. Columns represent module groups. Rows represent hierarchical clustering of enriched GO categories; those with an asterisk indicate a biological process, while all others are molecular functions. Color represents–log_10_ adjusted p-value.

In order to study the temporal regulation of the *Rsv3*-mediated ER mechanism, we performed co-expression clustering of DEGs. The 1128 DEGs found between the two cultivars at one or more time points between 2 and 8 hpi were clustered into different co-expressed modules using a model-based clustering approach, where a module is defined as a group of genes sharing similar expression profiles over time and are likely functioning in the same biological processes. Based on BIC and ICL criteria, we identified five modules that optimally explain the observed gene expression pattern; these modules consist of 85 (module-1), 198 (module-2), 383 (module-3), 170 (module-4), and 292 (module-5) DEGs. The expression profile for these modules was determined by averaging the expression levels of DEGs within each module (Fig 2A). The expression profiles for module-1, module-4, and module-5 were similar between L29 and Williams82, whereas those for module-2 and module-3 were highly divergent between the two cultivars. This divergence in their expression pattern was noticeable between 4 and 8 hpi, with a peak at 6 hpi. For module-5, despite similar expression patterns, the magnitude of difference between L29 and Williams82 was greater in Williams82 than in L29.

GO enrichment analyses of five co-expression modules showed significant enrichment of 47 biological processes (shown with asterisk) and molecular functions (Fig 2B) (S2 Table). The co-expression module-2 showed enrichment for several GO terms associated with ABA and auxin biosynthesis and signaling pathways (Fig 2B). The expression profile of this module showed a clear contrast between L29 and Williams82, with a maximum (4-fold) difference at 6 hpi, suggesting that ABA- and auxin-related processes were likely down-regulated in SMV-resistant L29 soybean between 4 and 8 hpi (Fig 2A). [15] found that ABA-mediated callose deposition in cell walls prevents intercellular virus movement in *Rsv3*-mediated ER in SMV-G5H inoculated L29 after 8 hpi. Callose deposition was not observed in SMV-G7 inoculated L29 (this study); however, Glyma.16152600 and Glyma.03G132700, both encoding beta-1,3-glucanases, were down-regulated at 6 hpi in L29. This is interesting as one of ABA’s defense strategies against viruses is inhibition of these proteins, which function to degrade callose [56]. The down-regulation in L29 of genes encoding callose degradation proteins provides further evidence that *Rsv3* begins mounting a defense as early as 6 hpi. Additionally, [15] showed elevated expressions of ABA and ABA responsive genes in SMV-G5H inoculated L29 leaves after 8 hpi. In contrast, we observed down-regulation of ABA responsive genes in SMV-G7 inoculated L29 leaves before 8 hpi, indicating changes in ABA signaling begin soon after inoculation.

Co-expression module-4 showed enrichment of several GO terms associated with jasmonic acid (JA) biosynthesis and signaling and ethylene (ET) biosynthesis (Fig 2B). Module-4 expression showed similar profiles between the two cultivars but average expressions were lower in L29 than in Williams82 at 4, 6, and 8 hpi, suggesting JA suppression may be required for *Rsv3*-mediated ER (Fig 2A). Suppression of JA pathway in *Rsv3*-mediated resistance was also reported in SMV-G5H inoculated L29 cultivar [56]. Though JA’s role in viral defense is not well understood, [43] observed that increased JA levels in soybean enhance susceptibility to BPMV. Interestingly, co-expression module-5 was enriched with genes associated with biological processes such as for syncytium formation (GO:0006949), cell wall modifications (GO:0009828, GO:0009831), cytokinin (CK) degradation (GO:0009823, GO:0019139), and cell growth (GO:0009826). Enrichment for these processes is indicative of virus interference with cell growth and metabolism. As for the expression profile of this module, it fluctuated drastically from 2 hpi to 8 hpi in Williams82 compared to the subtle shifts in L29. This may indicate greater changes in the activity of these biological processes in Williams82, which are perhaps associated with soybean susceptibility to SMV and stages of virus replication occurring as early as 4 hpi (Fig 2A).

For the enrichment in CK degradation, multiple genes encoding cytokinin dehydrogenases were up-regulated in L29 from 2 to 6 hpi, suggesting CK levels were reduced in L29 relative to Williams82. CKs function to promote cell proliferation and elongation, numerous developmental processes, and are known to have a role in viral resistance [53]. In Williams82, the large expression changes in genes involved in membrane activity, syncytium formation, cell wall loosening, and cell growth and modification are known to be associated with early and initial stages of the potyvirus infection process in susceptible hosts [57, 58]. In particular, syncytium formation is a biological process in which virus-infected cells fuse together to form enlarged multi-nucleated cells called syncytia [59]. The increase in gene products used to form syncytia, which are not known to occur in cells of potyvirus-infected plants, may reflect the initiation of virus replication in the susceptible host, Williams82, as it did not occur in L29. After all, potyviruses are known to form 6K2 membrane-bound vesicles that later form tubular structures and interact with host endoplasmic reticulum [60]. This response could have been facilitated by heightened CK levels in Williams82. Interestingly still, CKs can act synergistically with the SA signaling pathway, triggering its activation [53]. In fact, [61] proposed that CK levels might aid in determining the amplitude of SA-related immunity. Perhaps in the case of soybean *Rsv3*-mediated resistance, where it seems suppression of the SA pathway is required, this suppression is achieved through reduced CK levels.

Only single biological processes such as responses to sucrose starvation and absence of light were enriched for the co-expression module-1 and module-3, respectively, but the analyses of these modules will not be included in this study. We also analyzed gene family enrichment using an online tool, GenFam [47]. We found that some results are in agreement with the GO analysis. In particular, GenFam found that “Kunitz Trypsin Inhibitor (KTI) gene family” is enriched in module-2, whereas GO analysis showed (GO:0004866) endopeptidase inhibitor activity is also enriched in module-2. This result from GenFam is more specific than GO annotation because KTI is a specific type of endopeptidase inhibitor. Similarly, we also found “Expansin gene family” is enriched in module-5, whereas GO analysis showed (GO:0009828) plant-type cell wall loosening is also enriched in module-5. Although many factors might regulate plant-type cell wall loosening, the results from GenFam enrichment provide a more specific result suggesting expansin genes are the main gene family contributing to cell wall loosening in our experiment.

### Suppression of MYC2 transcription factor expression is important for *Rsv3*-mediated ER

Our network inference analysis identified candidate genes regulating gene expression in each module. Between the five network inference methods, a total of 654 interactions were identified between TF genes and the gene co-expression modules. No interaction was predicted by all five methods, but 56 interactions were predicted by four out of five methods (S3 Table). These 56 TF-module interactions were regulated by 49 TFs, indicating some TFs regulated more than one module, and all five modules were regulated by more than one TF. Because there could be an unknown number of false negatives (true interactions that were not supported by expression data) and false positives (interactions supported by expression data but not found in biological systems) in the predicted interactions, we chose to use bioinformatics approaches to validate our computational predictions. In the rest of this manuscript, we focused on the predicted interactions that are supported by homologous interactions in the model species, *A*. *thaliana*, and also analyzed the motif enrichment to compare with known motifs in *A*. *thaliana*.

When the 56 putative interactions were transformed to homologous *A*. *thaliana* interactions, comparison to the *A*. *thaliana* DAP-seq dataset validated 1732 TF-gene interactions, with 21 TFs and 819 genes (S4 Table). This translates to 25 TF-module interactions found from the network inferred 56 TF-module interactions (S5 Table). Further validation by motif sequence analysis discovered 20 enriched motifs in the five modules, with each module containing enrichment of one or more motifs (S6 Table). The identified motifs represent putative TF binding sites from which TFs can regulate the expression of target genes in each of the modules; this allowed us to identify TF families that may recognize and bind to the enriched motif sequences. From the 25 TF-module interactions validated with the *A*. *thaliana* DAP-seq data, we found nine interactions further validated by motif sequence analyses (Table 1). Still, though the *A*. *thaliana* DAP-seq dataset is large, it does not represent every interaction; therefore, we included three additional interactions from the inferred 56 TF-module interactions that were validated by motif enrichment only.

**Table 1:** *A*. *thaliana* and motif validated interactions.

TF Name	TF Family	Target Module	*A*. *thaliana* Homolog	MEME Motif Enrichment E-value	MEME Motif	DAP-seq Motif	DAP-seq Motif Similarity p-value
Glyma.07G060400	bZIP	1	AT2G46270	2.00E-20	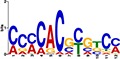	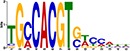	3.59E-04
Glyma.04G036700	MYB	2	AT3G50060	2.40E-19	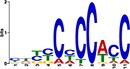	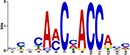	8.16E-04
Glyma.07G051500*	MYC2 (bHLH)	2	AT1G32640	9.30E-24	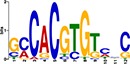	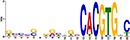	5.58E-05
Glyma.06G092000*	bHLH	3	AT5G65640	6.20E-05	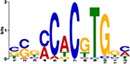	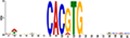	7.62E-05
Glyma.17G090500*	bHLH	4	AT4G20970	2.30E-04	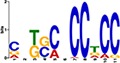	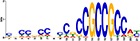	2.22E-04
Glyma.17G145300	ERF	4	AT5G47230	1.60E-02	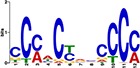	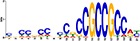	1.78E-06
Glyma.08G042100	MYB	4	AT1G25340	1.00E-18	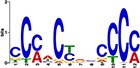	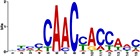	1.90E-05
Glyma.02G080200 Glyma.08G216600 Glyma.05G234600 Glyma.08G042100	ERF ERF MYB MYB	5	AT2G33710 AT5G25190 AT1G25340 AT1G25340	2.10E-11	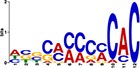	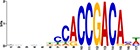	2.89E-04 4.24E-03
						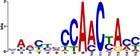	5.01E-06
Glyma.18G301500	NAC	5	AT5G13180	1.20E-33	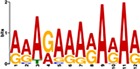	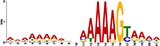	5.01E-06

Shown are putative TF-module interactions with their validation results from motif sequence analyses. MEME results show enriched motifs found in each module using promoter sequences of genes belonging to module. *A*. *thaliana* DAP-seq data was used to find motifs with high similarity to MEME motifs, which enabled identification of TFs that putatively recognize and bind the enriched MEME motifs discovered in each module.

*TFs with asterisks were validated by motif sequence analyses only.

**Fig 3:**
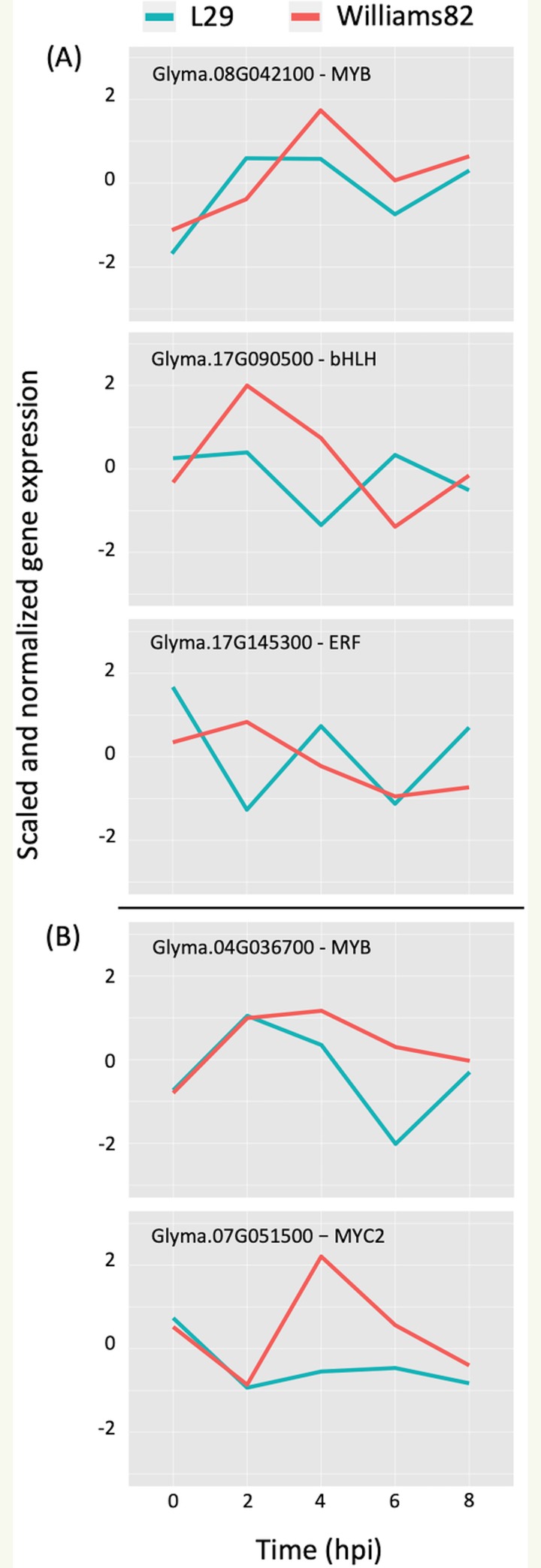
Comparison of normalized gene expression profiles of validated TFs in L29 and Williams82. (A) TFs predicted to regulate module-4. (B) TFs predicted to regulate module-2.

Motif sequence analyses showed that co-expressed genes in module-5 are regulated by NAC (NAM, ATAF1/2, and CUC), ERF (ethylene responsive factor) and/or MYB (myeloblastosis oncogene) TFs (Table 1). NAC TFs are major regulators of biotic and abiotic stress responses in plants. Several studies have shown the induction of NAC TFs upon virus infection and their essential role in basal defense and the innate plant immune system [62, 63]. This is consistent with the enrichment for genes associated with syncytium formation in module-5. The ERF TFs are well known to be involved in the regulation of disease resistance pathways [64, 65]. Their expression can be altered by pathogen attack and phytohormones like JA, SA, and ET [66]. Only one ERF TF gene (Glyma.17G145300) was found to regulate the JA responsive genes in module-4 (Fig 3A) (Table 1). The *A*. *thaliana* homolog of this gene encodes ERF5, which has been implicated as a regulator in the JA-mediated defense pathway [67]. The disparate expression profiles and putative function makes Glyma.17G145300 gene an ideal candidate for the differential regulation of JA-related processes found in module-4, which may lead to *Rsv3*-mediated ER response in soybean. Some genes in module-4 were also predicted to be regulated by a basic/helix-loop-helix (bHLH) TF (Glyma.17G090500) and a MYB TF (Glyma.08G042100) (Table 1). The bHLH TF (Glyma.17G090500) showed contrasting expression profiles between L29 and Williams82, with a two-hour lag in expression changes observed in Williams82 (Fig 3A). Another MYB TF (Glyma.04G036700) was also found to regulate genes in module-2, and its expression was significantly down-regulated in L29 at a 6 hpi (Fig 3B). MYBs are known to be involved in plant defense and stress responses [65]. In particular, MYB77, encoded by Glyma.04G036700 (the MYB regulating module-2), is associated with stress responses and is a modulator of auxin activity, of which module-2 was enriched with [68, 69].

The module-2 was significantly enriched for the G-box motif (“CACGTG”), which is specifically recognized by the bHLH TF superfamily, and our network happened to predict a bHLH (Glyma.07G051500) regulating module-2 (Table 1) [70, 71]. This TF was differentially expressed at 4 hpi with a log2 fold change of -2.30 in L29, showing it was triggered prior to the major transcriptional shift observed at 6 hpi. Comparison of its expression pattern revealed vastly different profiles, with a significant peak in expression in Williams82 (Fig 3B**)**. This gene was also identified as a putative resistance gene against a leaf-eating insect, the common cutworm, and similarly, its expression levels were also significantly lower at 4 hpi in the resistant line [72]. This suggests Glyma.07G051500’s activity is important in pathogen defense. The *A*. *thaliana* homolog (AT1G32640) of Glyma.07G051500 encodes a MYC-related transcriptional activator (MYC2) with a bHLH leucine zipper DNA binding domain [73].

*MYC2* is reported to condition resistance to insects and regulate ABA signaling, JA-responsive pathogen defense, oxidative stress response genes, and other TFs’ expressions, as well as negatively regulate its own expression [73–79]. Notably, *MYC2* is described as a “master switch” in modulating both positive and negative crosstalk between ABA and JA signaling [80]. As mentioned earlier, we found enrichment for both ABA- and JA-related processes in this study; thus MYC2, encoded by Glyma.07G051500, could be a key regulator in mediating the modular phytohormone responses observed with *Rsv3*-mediated ER. Interestingly, examination of the data from the study using avirulent SMV-G5H and virulent SMV-G7H strains on L29 [56] revealed that the *MYC2* gene Glyma.07G051500 as well as other *MYC2* genes were also exclusively expressed at low levels in L29 during *Rsv3*-mediated resistance. Interesting still, these are not the only instances where suppression of *MYC2* has been shown to promote resistance. In another RNA-seq experiment using near-isogenic soybean lines to study bacterial leaf pustule resistance, three genes encoding MYC2 TFs were expressed at low levels in the resistant line and predicted to be important for conditioning resistance [81]. In an even more striking genome-wide association study (GWAS) on soybean, the same *MYC2* gene (Glyma.07G051500) that was found in this study was identified as a putative resistance gene against the common cutworm where its expression was also significantly down-regulated in the resistant line [72]. Even in tomato, *MYC2* has been shown to regulate immunity via the JA pathway by coordinating a transcriptional cascade [82]. Taken together, these findings indicate that *MYC2* activity may be important in pathogen defense. In particular, it appears that suppression of its activity may in some cases promote resistance, which may be a consequence of its status as a master regulator, allowing it to efficiently suppress expression of targets exploited by pathogens. Because, perhaps by altering a master regulator’s expression, the expression of numerous downstream genes (some of which may be targets for pathogen exploitation) can be altered in such a way as to condition resistance. Whatever the case, the function of *MYC2* in relation to *Rsv3*-mediated ER poses an interesting subject for more research, as it may be responsible for many of the changes observed in ABA and JA signaling that are observed during *Rsv3* resistance [15, 56].

### Modular regulation of abscisic acid signaling and suppression of jasmonic acid signaling are features of *Rsv3*-mediated ER

We examined the gene targets of the *MYC2* (Glyma.07G051500) and *MYB* (Glyma.04G036700) TFs regulating module-2. In particular, we looked at genes involved in ABA, auxin, and defense processes (Table 2). All gene targets were down-regulated at 6 hpi in L29. Among the targets were genes encoding ABA and auxin responsive element-binding factors (ABFs, SAUR), ABI five-binding proteins (AFPs), type 2C protein phosphatases (PP2Cs), and MYB-like TFs (RVE1s).

**Table 2:** TF target genes in module-2 related to ABA and auxin processes and defense responses.

Target Gene	*A*. *thaliana* Homolog	Regulator TF	L29 Log2 Fold Change at 6hpi	Gene Symbol	Description
Glyma.07G074400	AT3G61220	MYB	-2.34	SDR1	(+)-neomenthol dehydrogenase
Glyma.09G218600	AT4G19230	MYB	-2.22	CYP707A1	Abscisic acid 8'-hydroxylase 1
Glyma.02G131700	AT1G49720	MYB, MYC2	-1.11	ABF1	Abscisic acid responsive element-binding factor 1
Glyma.06G040400	AT1G45249	MYB	-1.43	ABF2, AREB1	Abscisic acid responsive elements-binding factor 2
Glyma.15G105100	AT5G19140	MYB	-1.04	AILP1, ATAILP1	Aluminum induced protein with YGL and LRDR motifs
Glyma.09G005700	AT1G62300	MYB, MYC2	-1.56	-	At1g62300 protein (Fragment)
Glyma.09G219300	AT5G18050	MYB	-2.23	SAUR22	Auxin-responsive protein
Glyma.04G061500	AT5G25110	MYB, MYC2	-1.39	CIPK25, PKS25, SnRK3.25	CBL-interacting serine/threonine-protein kinase 25
Glyma.06G062100	AT5G25110	MYB	-1.97	CIPK25, PKS25, SnRK3.25	CBL-interacting serine/threonine-protein kinase 25
Glyma.20G241700	AT3G55120	MYB	-1.50	CHI1, CFI, TT5	Chalcone—flavonone isomerase 1
Glyma.16G194600	AT3G05200	MYB	-1.80	ATL6	E3 ubiquitin-protein ligase
Glyma.09G140700	AT3G05200	MYB	-1.72	ATL6	E3 ubiquitin-protein ligase
Glyma.07G060400	AT2G46270	MYB, MYC2	-1.56	GBF3	G-box binding factor 3
Glyma.12G117700	AT2G20570	MYB, MYC2	-1.11	GPRI1, GLK1	GBF's pro-rich region-interacting factor 1
Glyma.02G241000	AT5G17300	MYB, MYC2	-2.11	RVE1	Homeodomain-like superfamily protein
Glyma.13G152300	AT5G17300	MYB	-1.69	RVE1	Homeodomain-like superfamily protein
Glyma.14G210600	AT5G17300	MYB, MYC2	-1.78	RVE1	Homeodomain-like superfamily protein
Glyma.06G319600	AT1G33590	MYB, MYC2	-2.59	-	Leucine-rich repeat (LRR) family protein
Glyma.13G253300	AT1G09970	MYB	-1.39	-	Leucine-rich repeat receptor-like kinase
Glyma.20G054000	AT3G45140	MYB, MYC2	-1.11	LOX2	Lipoxygenase 2
Glyma.02G272700	AT5G20990	MYB	-1.08	-	Molybdopterin biosynthesis CNX1 protein
Glyma.01G060300	AT1G13740	MYB, MYC2	-2.12	AFP2	Ninja-family protein AFP2 (ABI five-binding protein 2)
Glyma.02G118500	AT1G13740	MYB, MYC2	-1.91	AFP2	Ninja-family protein AFP2 (ABI five-binding protein 2)
Glyma.18G267200	AT1G13740	MYB, MYC2	-1.60	AFP2	Ninja-family protein AFP2 (ABI five-binding protein 2)
Glyma.04G014000	AT3G18830	MYB	-1.62	PLT5	Polyol transporter 5
Glyma.13G076700	AT3G20770	MYB	-1.34	EIN3	Protein ETHYLENE INSENSITIVE 3
Glyma.20G051500	AT3G20770	MYB	-1.02	EIN3	Protein ETHYLENE INSENSITIVE 3
Glyma.19G069200	AT1G07430	MYB	-1.55	AIP1	Protein phosphatase 2C 3
Glyma.08G033800	AT4G26080	MYB	-1.09	ABI1	Protein phosphatase 2C 56
Glyma.02G086100	AT1G14790	MYB	-1.87	RDR1, RDRP1	RNA-dependent RNA polymerase 1

Shown are target genes, the TFs putatively regulating them, log2 fold change of target genes, and target genes' functions based on *A*. *thaliana* homologs.

We also examined JA- and defense-related gene targets of the *bHLH* (Glyma.17G090500), *ERF* (Glyma.17G145300), and *MYB* (Glyma.08G042100) TFs regulating the module-4 (Table 3). Most genes were expressed at low levels in L29, such as those involved in JA biosynthesis and a number of TFs; however, at 2 hpi, a few genes were up-regulated. These were Glyma.19G164600 encoding an MYB14 TF, and Glyma.12G114100 encoding an L-type lectin receptor kinase, which induces hydrogen peroxide production, cell death, and is required for resistance to oomycetes and fungal pathogens [83, 84]. Lastly, Glyma.11G139500 encoding another PP2C was also up-regulated in L29. This protein family was shown to be an essential signaling component of *Rsv3*-mediated ER against SMV, involved in inducing callose deposition via the ABA signaling pathway [15]. We found that differential regulation of *PP2C* genes begins as early as 2 hpi, suggesting the *Rsv3* resistance pathway is elicited almost immediately after inoculation.

**Table 3:** TF target genes in module-4 related to JA processes and defense responses.

Target Gene	*A*. *thaliana* Homolog	Regulator TF	L29 Log2 Fold Change	hpi	Gene Symbol	Description
Glyma.13G361900	AT1G15520	ERF	-1.05	4	ABCG40, PDR12, PDR9	ABC transporter G family member 40
Glyma.01G153300	AT4G19230	bHLH, ERF, MYB	-1.19	4	CYP707A1	Abscisic acid 8'-hydroxylase 1
Glyma.19G044900	AT3G25780	bHLH, ERF, MYB	-1.11	4	AOC3	Allene oxide cyclase 3
Glyma.17G007600	AT4G17230	bHLH	-1.72	4	-	AT4G17230 protein (Fragment)
Glyma.05G082400	AT5G66900	MYB	-2.43	6	MUD21.16	Disease resistance protein (CC-NBS-LRR class) family
Glyma.02G132500	AT4G34410	bHLH, MYB	-1.45	4	ERF109	Ethylene-responsive transcription factor 109
Glyma.15G078600	AT1G28480	bHLH, ERF	-1.08	4	GRXC9, GRX480, ROXY19	Glutaredoxin-C9
Glyma.11G038600	AT1G19180	MYB	-2.61	4	JAZ1	Jasmonate-zim-domain protein 1
Glyma.15G179600	AT1G19180	MYB	-1.69	4	JAZ1	Jasmonate-zim-domain protein 1
Glyma.12G114100	AT4G28350	bHLH, MYB	1.78	2	LECRK72, LECRKD	L-type lectin-domain containing receptor kinase
Glyma.13G030300	AT3G45140	bHLH, MYB	-1.68	6	LOX2	Lipoxygenase 2
Glyma.07G039900	AT1G17420	MYB	-1.13	4	LOX3	Lipoxygenase 3
Glyma.04G226700	AT4G35580	bHLH	-1.05	2	NTL9, CBNAC	NAC transcription factor-like 9
Glyma.06G138100	AT4G35580	bHLH	-1.01	2	NTL9, CBNAC	NAC transcription factor-like 9
Glyma.11G228100	AT2G40000	MYB, ERF	-1.19	6	HSPRO2	Nematode resistance protein-like
Glyma.11G139500	AT1G07630	bHLH, ERF, MYB	1.13	2	PLL5	Protein phosphatase 2C 4
Glyma.01G204400	AT1G74950	bHLH, ERF, MYB	-2.30	4	TIFY10B, JAZ2	Protein TIFY 10B
Glyma.09G145600	AT1G47890	MYB	-2.46	4	RLP7	Receptor-like protein 7
Glyma.07G189300	AT4G21440	bHLH, MYB	-1.62	4	MYB102	Transcription factor MYB102
Glyma.19G164600	AT2G31180	bHLH, MYB	2.57	2	MYB14	Transcription factor MYB14
Glyma.01G128100	AT2G38470	ERF	-2.49	4	WRKY33	WRKY transcription factor 33

Shown are target genes, the TFs putatively regulating them, log2 fold change of target genes, and target genes' functions based on *A*. *thaliana* homologs.

Between the differential regulation of several TFs and signaling molecules, such as the ABF, AFP, PP2C, and JAZ encoding genes in modules 2 and 4, it appears a complex transcriptional cascade is at work, finely regulating both ABA and JA signaling. Characteristically, ABA and JA are mutually antagonistic in a defense response [74, 85]; however, according to our results, this does not appear to be the case during the early hours of *Rsv3*-mediated resistance. Between 0 and 8 hpi, ABA- and JA-related genes were largely down-regulated in L29, indicating a signaling scheme divergent from the typical antagonistic relationship between ABA and JA. The purpose of this interaction is not clear, but certain components of their signaling pathways, such as ABFs in the ABA pathway, may be targets for viral exploitation and would thus require suppression in order to condition SMV resistance. For example, high *ABF1* expression was observed during *Sonchus yellow net virus* and *Impatiens necrotic spot virus* infection [86]; thus *ABF* suppression may also be important for escaping SMV infection. However, it seems some aspects of the ABA pathway must remain functional, as ABA accumulation was observed in *Rsv3*-mediated ER at 8 hpi and later [15]. This suggests the ABA signaling pathway may be modular in L29, with it first being silenced during the early hours post-inoculation (2–8 hpi) and then later re-activated (8 hpi). Evading viral exploitation may be the case for the JA pathway as well, as genes functioning in this pathway were mostly suppressed (4–8 hpi) in L29. This suppression was also observed in another *Rsv3* RNA-seq study at times even later than 8 hpi [56]. Even more, JA biosynthesis has been shown to increase susceptibility to some viruses in soybean [55]. Consequently, and unlike the modular regulation pattern found with the ABA pathway, it may be critical for the JA pathway to remain suppressed in order for *Rsv3*-mediated resistance to be conferred; such a condition would be worthwhile to investigate. Regardless, it appears that a finely regulated phytohormone network conditions *Rsv3*-mediated resistance via suppression of the JA pathway and modular regulation of the ABA signaling pathway. This carefully orchestrated network may help explain how *Rsv3*-mediated ER is able to swiftly coordinate a defense against SMV.

## Conclusion

In conclusion, we compared the transcriptomic response of two soybean varieties exhibiting susceptible and resistant phenotype to SMV-G7 strain and constructed gene regulatory networks to identify key genes and transcription factors that regulate the *Rsv3*-mediated ER mechanism in soybean. Our findings suggest that the *Rsv3*-mediated ER response is initiated early after inoculation once the fate of susceptibility or resistance to SMV is determined. The *Rsv3*-mediated ER response appears to largely involve differential regulation of various phytohormone pathways, suggesting phytohormone signaling to be fundamental in *Rsv3*-mediated resistance. In particular, early suppression of SA, CK, ABA, and JA pathways and the interplay of ABA and JA pathways may be essential. Different TFs, MYC2 in particular, were found to regulate these signaling events possibly via down-regulation of numerous genes to evade viral exploitation in the SMV-resistant cultivar L29 (*Rsv3*-genotype). While experimentation is needed for further confirmation, our analyses predict potential candidate genes for hypothesis-driven experiments. Overall, this study offers new insights into the unique and intricate regulation of the *Rsv3*-mediated ER response to *Soybean mosaic virus*.

## Supporting information

S1 Table Log2 fold change for differentially expressed genes for time pair comparisons. (XLSX)

S2 Table Gene ontology enrichment analysis (GO terms with padj < .05 only). (XLSX)

S3 Table Interactions predicted by four out of five network inference methods. (XLSX)

S4 Table Putative TF-gene interactions supported by orthologous interactions found in *A*. *thaliana*. (XLSX)

S5 Table Putative TF-module interactions supported by orthologous interactions found in *A*. *thaliana*. (XLSX)

S6 Table Motif enrichment analysis of co-expression modules and transcription factors recognizing motif sequences. (XLSX)
